# Evaluating the Utility of a New Pathogenicity Predictor for Pediatric Cardiomyopathy

**DOI:** 10.1155/2023/8892833

**Published:** 2023-10-27

**Authors:** Alyssa L. Rippert, Sarah Trackman, Danielle Burstein, J. William Gaynor, Heather Griffis, Christine Seymour, Rebecca Ahrens-Nicklas

**Affiliations:** ^1^Division of Human Genetics, Children's Hospital of Philadelphia, Pennsylvania, USA; ^2^Rutgers, The State University of New Jersey, New Brunswick, NJ, USA; ^3^Division of Pediatric Cardiology, University of Vermont Medical Center, Burlington, VT, USA; ^4^Division of Cardiothoracic Surgery, Children's Hospital of Philadelphia, Philadelphia, PA, USA; ^5^Department of Biomedical and Health Informatics, Children's Hospital of Philadelphia, Pennsylvania, USA

## Abstract

Pediatric cardiomyopathy (CM) has significant childhood morbidity and mortality which is caused by both genetic and environmental factors. Previous research has focused on identifying genetic variants in pediatric CM for diagnostic purposes, but not for risk stratification. The current study was modeled after previous work which showed an association between CardioBoost-classified disease-causing variants and an increased risk for severe clinical outcomes in adults with CM to assess if the same association is true in pediatric CM. This was a retrospective, single-center cohort study that evaluated outcomes in pediatric CM patients who were evaluated by the Children's Hospital of Philadelphia (CHOP). CardioBoost (CB) scores were generated for these patients, and scores were categorized as ≤0.1, 0.1-0.9, and ≥0.9. Composite endpoint was freedom from a major adverse cardiac event (MACE). 104 patients were included in the final analysis. 32 (31%) had DCM, 45 (43%) had HCM, and 27 (26%) had other CM. There was no significant association between CB score and clinical outcome in pediatric CM patients. Overall, this study highlights the continued deficits in variant interpretation for pediatric CM. We recommend using caution when applying this tool to stratify clinical outcomes in the pediatric population.

## 1. Introduction

Pediatric cardiomyopathy (CM) is a rare condition with an incidence of around 1 in 100,000 individuals. Pediatric cardiomyopathies include dilated (DCM, 55-60%), hypertrophic (HCM, 35-40%), restrictive (2-5%), left ventricular noncompaction (LVNC, <1%), and arrhythmogenic right ventricular cardiomyopathy (ARVD, <1%). Cardiomyopathies represent a wide phenotypic spectrum ranging from mild cardiac involvement to severe cardiac dysfunction [1]. Pediatric CM can cause significant morbidity and mortality in affected children and may progress to congestive heart failure, the need for advanced heart failure therapy, or cardiac death [2]. Around 6% of pediatric CM patients experience sudden cardiac death [3], and almost 50% of pediatric DCM patients progress to heart transplant or death within 5 years of initial diagnosis [4].

The causes of CM are diverse and include genetic variants, infectious agents, environmental factors, neuromuscular disease, and inherited metabolic disorders [1]. Pathogenic variants are identified in 50-65% of patients with pediatric HCM [5], 70% of patients with adult familial HCM [6], and 40-50% of patients with adult DCM [7]. Genetic testing has become an integral part of patient care for both children and adults with CM. Genetic testing can help confirm a clinical diagnosis, guide management decisions, and aid in familial risk assessment [8, 9]. Previous research in pediatric CM has focused on identifying pathogenic variants for diagnostic purposes. However, the presence of a pathogenic variant alone is insufficient to predict clinical outcomes and adverse events in pediatric cardiomyopathy [10]. There is limited research evaluating the relationship between variants and clinical outcomes.

With advances in next-generation sequencing, an increasing number of genetic variants, both pathogenic and variants of uncertain significance (VUS), are emerging as potential causes of CM [10]. Historically, VUS have not been considered when evaluating genotype-outcome associations. However, a recent study suggested that increased variant burden, including VUS, was associated with worse clinical outcomes in patients with pediatric DCM. Thus, consideration of both pathogenic variants and VUS as potential risk modifiers may help stratify risk for adverse clinical outcomes [10]. Despite this, interpretation of VUS and rare genetic variants still represent a significant challenge in clinical genetic testing for CM. Accurate discrimination between benign and pathogenic variation remains essential for proper management of patients with pediatric CM [11].

Determining the clinical significance of a variant is a complex process that involves gathering a multitude of data from a combination of resources. Computational prediction of variant pathogenicity is integrated as one line of supporting evidence to assess the clinical significance of a variant [12]. Existing genome-wide machine learning tools learn from large-scale data over the entire genome. However, they are less accurate and less specific than disease-specific variant classifiers [13]. CardioBoost is a disease-specific variant classifier that estimates the probability of pathogenicity for rare missense variants in inherited cardiomyopathies and arrhythmias. CardioBoost outputs a continuous probability of pathogenicity that is directly interpretable by the user. CardioBoost has been shown to outperform existing genome-wide tools in classification performance, prediction of disease association, and stratification of patient outcomes. While it is important to recognize that CardioBoost scores are one line of evidence in variant classification, variants classified as disease-causing by CardioBoost are associated with both disease status and an increased risk of severe clinical outcomes in adults with HCM [13].

The purpose of this study is to determine whether CardioBoost can be used to better stratify disease severity and clinical outcomes in patients with pediatric CM. Genetic risk stratification of pediatric CM could have significant clinical implications including individualizing cardiac treatments and providing prophylactic interventions before adverse events occur.

## 2. Materials and Methods

### 2.1. Study Cohort

This is a retrospective, single-center cohort study that evaluated outcomes in pediatric patients (age < 21 years at diagnosis) with a history of cardiomyopathy (including DCM, HCM, restrictive, LVNC, or ARVD) who were evaluated by the Children's Hospital of Philadelphia (CHOP) Cardiomyopathy Program between January 1, 2010, and August 31, 2018. Patients were excluded from the analysis if there were insufficient records for review or if they had negative phenotype on screening. Cardiomyopathy was categorized as DCM, HCM, or other (restrictive, LNVC, or ARVD). Data, including demographic information, cardiomyopathy diagnosis, cardiac surgical information, physical examination, cardiac and metabolic laboratory data, cardiac imaging results, and genetic test results, were abstracted from the medical record. The Children's Hospital of Philadelphia Institutional Review Board (IRB #18-015616) approved this study with a waiver of consent. This work was previously published on ProQuest as a Master's thesis for ST [14].

ACMG classification was determined by a clinical geneticist (RAN) for each variant based on data available at time of data abstraction [12]. CardioBoost scores were generated for patients who meet study criteria and CardioBoost eligibility. CardioBoost can output predictions for rare (allele frequency < 0.1% in Gnomad) missense variants in a subset of genes associated with CM (*CACTC1*, *DES*, *GLA*, *LAMP2*, *LMNA*, *MYBP3*, *MYH7*, *MYL2*, *MYL3*, *PLN*, *PRKAG2*, *PTPN11*, *SCN5A*, *TNNI3*, *TNNT2*, and *TPM*). For patients with more than 1 variant with a CardioBoost score, the maximum score was utilized. Scores were then categorized as ≤0.1, 0.1–0.9, and ≥0.9 based on distribution of scores and CardioBoost's predefined categorizations. A CB score of ≤0.1 indicated a predicted pathogenicity of benign/likely benign. CB score of 0.1-0.9 indicated a predicted pathogenicity of VUS. CB score of ≥0.9 indicated a predicted pathogenicity of likely pathogenic/pathogenic. Concordance between ACMG classification and CB classification was evaluated based on these three categories of classification (benign/likely benign, VUS, and likely pathogenic/pathogenic).

### 2.2. Statistical Analysis

The composite endpoint was freedom from major adverse cardiac event (MACE), defined as internal cardioverter defibrillator (ICD) implantation, myectomy, mechanical circulatory support (ventricular assist device (VAD)) or extracorporeal membrane oxygenation (ECMO), cardiac transplant, and aborted cardiac arrest or death. The need for transplant evaluation with the decision to not list due to patient or family request was included in the composite endpoint. Secondary endpoints included freedom from individual MACE endpoints.

Demographic, clinical, imaging, and genetic variables were summarized using standard descriptive statistics. Categorical variables were reported as counts (with percentages), and nonnormally distributed continuous data were reported as median (with interquartile range (IQR)). Chi-square, Fisher's exact, Wilcoxon's rank-sum, and Kruskal-Wallis's tests were used to explore the relationship between CardioBoost score and patient characteristics. Bivariate odds ratios (ORs) and 95% confidence intervals (95% CIs) are reported for overall MACE and each component of MACE. The Kaplan-Meier curves are presented as time to MACE overall and stratified by CardioBoost scores ≤ 0.1, 0.1–0.9, and ≥0.9. Models are presented for the association between CardioBoost score and outcome via logistic regression and are adjusted for known confounders of outcome (race, sex, and age at first encounter). Logistic regression was performed as only date at MACE was available, not individual dates at each endpoint. Statistical significance is determined as a two-tailed *p* value ≤ 0.05. Analyses were conducted utilizing Stata version 17.

## 3. Results and Discussion

### 3.1. Patient Characteristics in Overall Cohort

**Figure 1:**
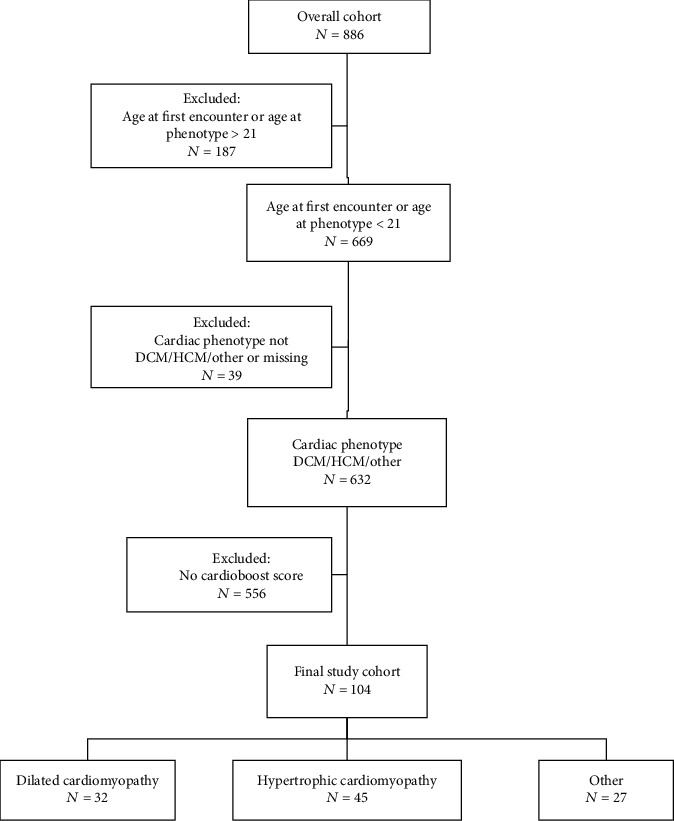
Flow chart of study population selection. Subjects were excluded from the final analysis if they were older than 21 at the time of first encounter or positive phenotype; had a congenital heart disease (CHD) other than dilated cardiomyopathy (DCM), hypertrophic cardiomyopathy (HCM), or other (restrictive, LNVC, or ARVD); or had a variant that was not interpretable by CardioBoost.

**Figure 2:**
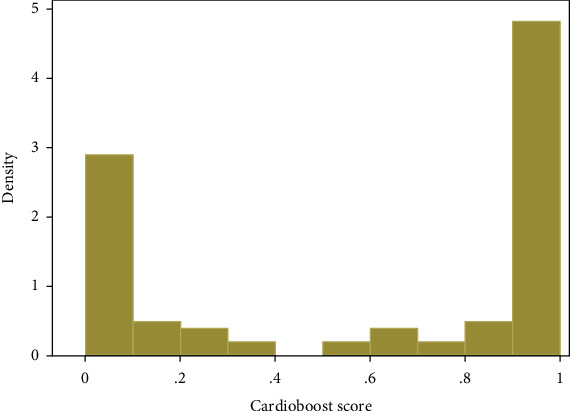
Histogram displays the bimodal distribution of CardioBoost scores across the overall cohort.

104 patients were included in the final analysis, of which 32 (31%) had DCM, 45 (43%) had HCM, and 27 (26%) had other (Figure 1 and Table 1). 71 (68%) were male and 33 (32%) were female (Table 1). 66 (64%) were white and 38 (36%) were nonwhite. The median age at first encounter was 5.3 (0.2–14.7) years, median age at last encounter was 14.9 (5.0–18.0) years, median age at positive phenotype was 5.7 (0.1–14.4) years, and median age at MACE was 10.4 (0.5–15.0) years. The median follow-up was 3.4 (1.0–6.0) years. The CardioBoost score distribution was bimodal (Figure 2); median CardioBoost score was 0.843 (0.055–0.99).

### 3.2. Genetic Testing and Variant Distribution

The most common genetic test was a cardiomyopathy panel, which was performed in 81% overall, 88% of DCM cases, 71% of HCM cases, and 89% of other cases (Table 2). Whole exome sequencing was performed in 7% overall, 6% of DCM cases, 4% of HCM cases, and 11% of other cases. Whole exome sequencing with mitochondrial sequencing was performed in 5% overall, 9% of DCM cases, 4% of HCM cases, and 0% of other cases. Patients with HCM were more likely to have targeted testing for a known familial variant (*p* = 0.001), which was performed in 14% overall, 6% of DCM cases, 27% of HCM cases, and 4% of other cases.

**Figure 3:**
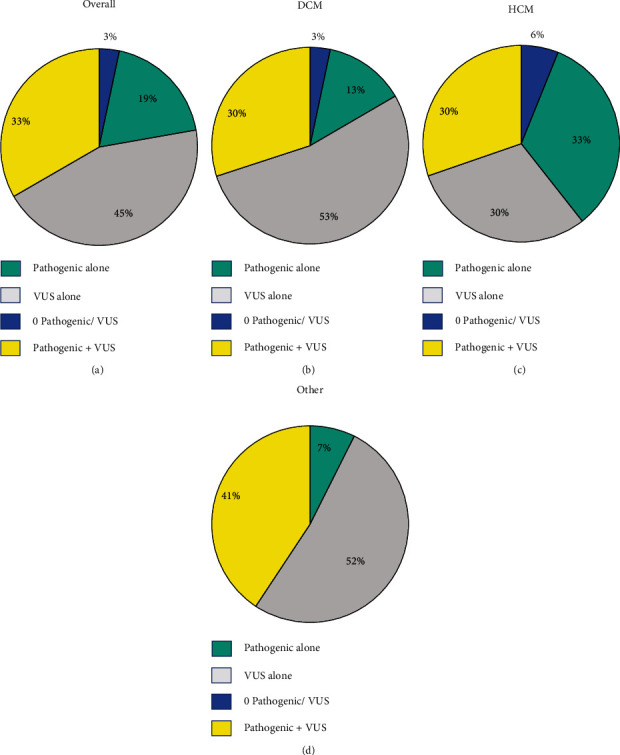
The distribution of genetic variants is presented across the overall cardiomyopathy cohort (a), the dilated cardiomyopathy cohort (b), the hypertrophic cardiomyopathy cohort (c), and the other cohort (d).

The distribution of genetic variants by ACMG classification was not significantly different across phenotypes (Figure 3). Around half of the overall cohort had at least one pathogenic variant, and around three-quarters had at least one VUS. The HCM cohort had the highest proportion of pathogenic variants, and the other cohort had the highest proportion of VUS. Inheritance of variants was most often unknown but was not significantly different across CM phenotypes.

### 3.3. Patient Characteristics by CardioBoost Score

Table 3 compares patient characteristics between those with CardioBoost (CB) scores ≤ 0.1, 0.1–0.9, and ≥0.9. The final study cohort had 16 scores ≤ 0.1, 14 scores 0.1-0.9, and 74 scores ≥0.9. The distribution of CB score was significantly related to race/ethnicity (*p* = 0.025) with a higher proportion of CB score ≥ 0.9 in white than nonwhite participants (71.6% versus 28.4%). The distribution of CB score was also significantly related to sex (*p* = 0.023) with a higher proportion of CB score ≥ 0.9 in male than female participants. There was no significant difference between age of first encounter and age at positive phenotype based on CB score. The most common reason for presentation for those with CB score ≤ 0.1 was heart murmur (44%). The most common reason for presentation for those with CB scores 0.1-0.9 and ≥0.9 was heart failure (50% and 38%, respectively). The distribution of CB score was significantly related to the presentation for family screening (*p* = 0.037) with a higher proportion of CB score ≥ 0.9 compared to other types of presentation. There was no significant difference in family history based on CB score.

### 3.4. CardioBoost Score and ACMG Classification Concordance

ACMG variant classification as performed by a clinical geneticist and CB classification were concordant in 60/131 (49%) and discordant in 62/131 (52%) of unique variants identified in this cohort (Supplemental Table 1). Half (31/62) of the discordant classifications were variants classified by CB as benign/likely benign and ACMG as VUS. Conversely, 3/62 (4.8%) variants were classified by CB as VUS and ACMG as benign/likely benign. Approximately one-quarter (16/62, 25.8%) of variants were classified by CB as pathogenic and ACMG as VUS. A CB classification of VUS and ACMG classification of likely pathogenic/pathogenic were seen in 11/62 (17.7%) of variants. Finally, there was one variant (*GLA* c.1088G>A, p.Arg363His) with a CB classification of benign/likely benign and ACMG classification of likely pathogenic/pathogenic. This variant is a well-described recurrent variant identified in individuals with Fabry's disease [15], highlighting the possible limited utility of CB classification for nonsarcomeric variants.

### 3.5. CardioBoost Score and Clinical Outcomes

**Figure 4:**
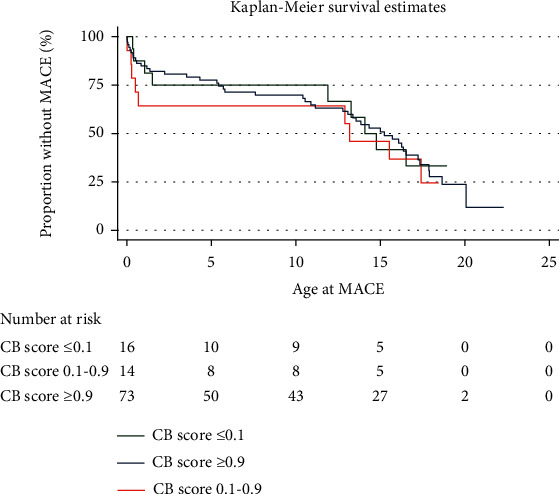
The Kaplan-Meier survival estimates for freedom from MACE are shown for patients in the overall cohort, stratified by CardioBoost score ≤ 0.1, 0.1-0.9, or ≥0.9. The composite endpoint MACE is defined as ICD implantation, myectomy, VAD, ECMO, cardiac transplant, transplant evaluation, aborted cardiac arrest, or death.

The composite MACE endpoint occurred in 61% of the overall cohort. The most common MACE were transplant (19%), ICD insertion (primary prevention) (14%), death (13%), and VAD implant (13%). A CB score of 0.1-0.9 (*p* = 0.654) or ≥9 (*p* = 0.736) was not significantly associated with the composite MACE endpoint (Table 4 and Figure 4) or any individual MACE endpoint (Table 4). There was no significant difference in survival estimates for freedom from MACE based on CB score (Figure 4).

## 4. Conclusions

This study did not identify a relationship between CB score and clinical outcome in patients with pediatric CM. Our study was modeled after a recent study by Zhang et al. that evaluated the ability of CB to stratify outcomes in a cohort of 803 adults with HCM [13]. They found that variants classified as disease-causing by CB were associated with a 21% increased risk of severe adverse outcomes by age 60. There are several possible explanations for why this finding was not replicated in a pediatric cohort, including a smaller sample size and a shorter length of follow-up. Pediatric CM is more rare than adult CM, with an incidence of around 1/100,000 [1] children compared to 1/500 adults [15]. This distinction is reflected in our respective sample sizes. It is also important to consider that pediatric and adult CM are different conditions with distinct pathological mechanisms [16]. A recent study investigating the genetic architecture of pediatric CM found that although genes identified from adult CM studies provide clinical value, it is still important to consider additional genes, as well as multigenic inheritance models, in the pediatric setting [16]. Before adjusting for confounding variables, we found that CB score ≥ 0.9 was protective for death. However, after adjusting for known confounders of outcome (race, sex, and age at first encounter), we did not identify any associations between CB score and clinical outcome.

A limitation of this study was the length of follow-up. Our median age at last encounter was 14.9 years; therefore, we did not capture adverse events that may have occurred in later adolescence or adulthood. It is also important to highlight the limitations of using CB in the pediatric setting. First, the CB tool was trained with adult data and might not perform as effectively for pediatric disease. In addition, CB scores are only available for 16 CM-related genes. Our cohort began at 886 patients, and 556 were excluded from the final analysis because they did not have a variant interpretable by CB. Future studies can consider following patients for a longer period to include more longitudinal pediatric data. In addition, multicenter evaluation of patients with pediatric CM could increase the sample size and generalizability of the study. The addition of new genes to CB may also allow for a larger sample size and help improve the clinical utility of the tool. Due to the intrinsic differences in the genetic architecture of adult and pediatric CM, it may be beneficial to create a variant classifier that is specifically designed for pediatric CM. Finally, concordance between ACMG and CB classification was only seen in 50% of unique variants identified in this cohort. This highlights the importance of multiple lines of evidence and rigorous application of ACMG classification guidelines for proper interpretation of these variants [12]. Overall, this study speaks to the continued deficits in variant interpretation for pediatric cardiomyopathy, and we recommend using caution when applying CardioBoost to stratify clinical outcomes in this population.

## Figures and Tables

**Table 1:** Demographics and clinical characteristics.

Sex	*N* (%)
Male	71 (68.3%)
Female	33 (31.7%)
Race	*N* (%)
White	66 (63.5%)
Nonwhite	38 (36.5%)
CM type	*N* (%)
DCM	32 (30.8%)
HCM	45 (43.3%)
Other	27 (26.0%)
	median (IQR)
Age at first encounter (year)	5.3 (0.2, 14.7)
Age at last encounter (year)	14.9 (5.0, 18.0)
Follow-up (year)	3.4 (1.0, 6.0)
Age at positive phenotype (year)	5.7 (0.1, 14.4)
Age at MACE (year)	10.4 (0.5, 15.0)
CardioBoost score	0.843 (0.055, 0.99)

**Table 2:** Genetic testing by cardiomyopathy cohort.

	CM type				
	Total (*n* = 104)	DCM (*n* = 32)	HCM (*n* = 45)	Other (*n* = 27)	*p* value
Genetic testing					
Cardiomyopathy panel	84 (80.8%)	28 (87.5%)	32 (71.1%)	24 (88.9%)	0.103
Whole exome sequencing	7 (6.7%)	2 (6.3%)	2 (4.4%)	3 (11.1%)	0.540
Whole exome sequencing+mitochondrial sequencing	5 (4.8%)	3 (9.4%)	2 (4.4%)	0 (0.0%)	0.320
Microarray	11 (10.6%)	7 (21.9%)	3 (6.7%)	1 (3.7%)	0.063
Targeted testing	14 (13.5%)	2 (6.3%)	12 (26.7%)	0 (0.0%)	0.001
Noonan panel	4 (3.8%)	0 (0.0%)	2 (4.4%)	2 (7.4%)	0.284
Inheritance					
Inherited	16 (15.4%)	3 (9.4%)	9 (20.0%)	4 (14.8%)	0.483
De novo	3 (2.9%)	2 (6.3%)	1 (2.2%)	0 (0.0%)	0.466
Unknown	61 (58.7%)	20 (62.5%)	21 (46.7%)	20 (74.1%)	0.070

**Table 3:** Patient characteristics by CardioBoost score.

	CardioBoost (CB) score			
	CB score ≤ 0.1	CB score 0.1-0.9	CB score ≥ 0.9	*p* value
Demographics				
Male	15 (93.8%)	7 (50.0%)	49 (66.2%)	0.023
Female	1 (6.3%)	7 (50.0%)	25 (33.8%)	
White	7 (43.8%)	6 (42.9%)	53 (71.6%)	0.025
Nonwhite	9 (56.3%)	8 (57.1%)	21 (28.4%)	
Age at first encounter (year)	1.1 (0.0, 11.8)	9.9 (0.0, 15.6)	6.5 (0.3, 14.8)	0.522
Age at last encounter (year)	17.6 (3.5, 16.5)	13.6 (2.8, 17.3)	14.0 (5.0, 17.8)	0.657
Follow-up (year)	3.3 (1.1, 16.5)	2.3 (0.3, 3.0)	3.8 (1.2, 6.0)	0.153
Age at positive phenotype (year)	1.1 (0.0, 10.8)	9.9 (0.0, 15.6)	8.4 (0.3, 14.8)	0.430
Presentation				
Heart failure	5 (31.3%)	7 (50.0%)	28 (37.8%)	0.562
Family screening	0 (0.0%)	1 (7.1%)	17 (23.0%)	0.037
Clinical screening	2 (12.5%)	1 (7.1%)	1 (1.4%)	0.071
Cardiac arrest	0 (0.0%)	2 (14.3%)	6 (8.1%)	0.347
Arrhythmia	1 (6.3%)	1 (7.1%)	1 (1.4%)	0.199
Chest pain	0 (0.0%)	0 (0.0%)	2 (2.7%)	1.000
Murmur	7 (43.8%)	2 (14.3%)	13 (17.6%)	0.074
Syncope	1 (6.3%)	1 (7.1%)	4 (5.4%)	1.000
Abnormal EKG	0 (0.0%)	0 (0.0%)	1 (1.4%)	0.496
Other	1 (6.3%)	0 (0.0%)	1 (1.4%)	1.000
Family history (FH)				
Positive FH	4 (25.0%)	5 (35.7%)	36 (48.6%)	0.181
First degree relative with a positive phenotype	2 (12.5%)	3 (21.4%)	25 (33.8%)	0.206
FH of sudden cardiac death	1 (6.3%)	1 (7.1%)	14 (18.9%)	0.384

**Table 4:** Association of CardioBoost score and clinical outcomes adjusted for race/ethnicity (nonwhite vs. white), sex (male vs. female), and age at first encounter. Reference group is ≤0.1. n/a = model was not able to run given cell size.

	OR (95% CI) CB ≤ 0.1 vs. 0.1-0.9	*p* value	OR (95% CI) CB ≤0.1 vs. ≥0.9	*p* value	Event rate
MACE	1.76 (0.35, 8.76)	0.491	1.78 (0.52, 6.05)	0.358	63 (60.6%)
Death	0.89 (0.14, 5.88)	0.907	0.22 (0.04, 1.17)	0.076	13 (12.5%)
Transplant	1.09 (0.17, 7.09)	0.931	1.01 (0.23, 4.39)	0.992	20 (19.2%)
ECMO	n/a	—	n/a	—	5 (4.8%)
VAD	n/a	—	n/a	—	13 (12.5%)
Aborted cardiac arrest	n/a	—	n/a	—	12 (11.5%)
ICD insertion (primary prevention)	0.33 (0.03, 3.80)	0.375	0.69 (0.17, 2.80)	0.602	15 (14.4%)
ICD insertion (secondary prevention)	n/a	—	n/a	—	3 (2.9%)
Myectomy	n/a	—	n/a	—	4 (3.8%)
Transplant evaluation	n/a	—	n/a	—	7 (6.7%)

## Data Availability

Data supporting this work is available upon request. Clinical data sets have been deidentified.

## Supplementary Materials

Supplementary Materials Supplemental Table 1: comprehensive details for each variant included in this analysis.
