# Supplementary material for: Evaluating the Utility of a New Pathogenicity Predictor for Pediatric Cardiomyopathy
Source: Hum Mutat. 2023 Oct 27;2023:8892833. doi: 10.1155/2023/8892833 (PMC11919062; doi:10.1155/2023/8892833)
Supplement: Supplementary Materials — Supplemental Table 1: comprehensive details for each variant included in this analysis. [file 8892833.f1.docx]

| Study ID | Gene or Chromosome | Variant | Genomic location (GRCh38) | ClinVar ID (if present) | gnomAD allele frequency (if present) | Sift | Polyphen | Cardioboost Score | Cardioboost classification | Final Classification | Concordance? |
| --- | --- | --- | --- | --- | --- | --- | --- | --- | --- | --- | --- |
| 371 | ACTC1 | c.1039A>G, p.Ile347Val | 15:34790507 | NM_005159.4(ACTC1):c.1039A>G | N/A |  |  | 0.56192 | VUS | VUS | Yes |
| 706 | ACTC1 | c.806T>C, p.Ile269Thr | 15:34792092 | NM_005159.4(ACTC1):c.806T>C | N/A |  |  | 0.80327 | VUS | Likely pathogenic | No |
| 426 | DES | c.28C>A, p.Arg10Ser | 2:219418490 | NM_001927.3(DES):c.28C>A | N/A |  |  | 0.01453 | Benign/LB | VUS | No |
| 582 | DES | c.376G>T, p.Val126Leu | 2:219418838 | NM_001927.3(DES):c.376G>T | N/A |  |  | 0.98875 | Path/LP | VUS | No |
| 848 | DES | c.656C>T, p.Thr219Ile | 2:219420267 | NM_001927.3(DES):c.656C>T | 0.0005722 | deleterious | prob damaging | 0.04246 | Benign/LB | VUS | No |
| 382 | GLA | c.352C>T, p.Arg118Cys | X:101403828 | NM_000169.2(GLA):c.352C>T | 0.0003954 | deleterious | poss damaging | 0.1717 | VUS | VUS | Yes |
| 413 | GLA | c.376A>G, p.S126G | X:101401803 | NM_000169.2(GLA):c.376A>G | 0.0004248 | deleterious | benign | 0.08189 | Benign/LB | Likely benign | Yes |
| 482 | GLA | c.1088G>A, p.Arg363His | X:101398011 | NM_000169.2(GLA):c.1088G>A | 0.0001325 | tolerated | benign | 0.002263 | Benign/LB | Pathogenic | No |
| 614 | LAMP2 | c.32G>T, p.Gly11Val | X:120469138 | NM_013995.2(LAMP2):c.32G>T | N/A |  |  | 0.03648 | Benign/LB | VUS | No |
| 136 | LMNA | c.107A>C, p.Gln36Pro |  |  |  |  |  | 0.27833 | VUS | Likely pathogenic | No |
| 439 | LMNA | c.1685T>A, p.Leu562His | 1:156137730 | NM_170707.3(LMNA):c.1685T>A | N/A |  |  | 0.01436 | Benign/LB | VUS | No |
| 745 | LMNA | c.1718C>T, p.Ser573Leu | 1:156138507 | NM_170707.3(LMNA):c.1718C>T | 0.0001046 | deleterious, low confidence | benign | 0.00155 | Benign/LB | VUS | No |
| 837 | LMNA | c.1930C>T, p.R644C | 1:156138719 | NM_170707.3(LMNA):c.1930C>T | 0.001166 | deleterious, low confidence | poss damaging | N/A | N/A | VUS | N/A |
| 3 | MYBPC3 | c.3323A>C, p.Lys1108Thr | 11:47333201 | NM_000256.3(MYBPC3):c.3323A>C | 0.0001117 | deleterious | prob damaging | 0.27831 | VUS | VUS | Yes |
| 70 | MYBPC3 | c.1504C>T, p.Arg502Trp | 11:47342698 | NM_000256.3(MYBPC3):c.1504C>T | 0.0000977 | deleterious | poss damaging | 0.93359 | Path/LP | Pathogenic | Yes |
| 72 | MYBPC3 | c.833G>A, p.G278E | 11:47347669 | NM_000256.3(MYBPC3):c.833G>A | 0.004183 | tolerated | benign | N/A | N/A | Benign | N/A |
| 117 | MYBPC3 | c.451G>A, p.Asp151Asn | 11:47350068 | NM_000256.3(MYBPC3):c.451G>A | 0.00001396 | tolerated | benign | 0.01047 | Benign/LB | VUS | No |
| 194 | MYBPC3 | c.1484G>A, p.Arg495Gln | 11:47342718 | NM_000256.3(MYBPC3):c.1484G>A | 0.00004187 | deleterious | poss damaging | 0.98421 | Path/LP | Pathogenic | Yes |
| 202 | MYBPC3 | c.3683G>C, p.Arg1228Pro | 11:47332203 | NM_000256.3(MYBPC3):c.3683G>C | N/A |  |  | 0.15963 | VUS | VUS | Yes |
| 234 | MYBPC3 | c.2914C>T, p.Arg972Trp | 11:47334002 | NM_000256.3(MYBPC3):c.2914C>T | 0.0009349 | deleterious | benign | 0.02142 | Benign/LB | Likely benign | Yes |
| 254 | MYBPC3 | c.2498C>T, p.A833V | 11:47337495 | NM_000256.3(MYBPC3):c.2498C>T | 0.007396 | tolerated | prob damaging | N/A | N/A | Benign | N/A |
| 358 | MYBPC3 | 2828G>A, R943Q | 11:47335119 | NM_000256.3(MYBPC3):c.2828G>A | 0.00003489 | deleterious | prob damaging | 0.05609 | Benign/LB | VUS | No |
| 378 | MYBPC3 | c.1813G>A, p.Asp605Asn | 11:47341222 | NM_000256.3(MYBPC3):c.1813G>A | 0.0001954 | tolerated | benign | 0.06384 | Benign/LB | VUS | No |
| 454 | MYBPC3 | c.2308G>A, p.Asp770Asn | 11:47338520 | NM_000256.3(MYBPC3):c.2308G>A | N/A |  |  | 0.72196 | VUS | Pathogenic | No |
| 454 | MYBPC3 | c.1519G>A, p.Gly507Arg | 11:47342683 | NM_000256.3(MYBPC3):c.1519G>A | 0.001731 | deleterious | prob damaging | 0.169 | VUS | Likely benign | No |
| 540 | MYBPC3 | c.961G>A, p.V321M | 11:47346336 | NM_000256.3(MYBPC3):c.961G>A | 0.0002442 | deleterious | poss damaging | 0.67618 | VUS | VUS | Yes |
| 582 | MYBPC3 | c.461t>c, p.Ile154Thr | 11:47350058 | NM_000256.3(MYBPC3):c.461T>C | 0.0001885 | tolerated | benign | 0.01671 | Benign/LB | VUS | No |
| 617 | MYBPC3 | c.3004C>T, p.Arg1002Trp | 11:47333743 | NM_000256.3(MYBPC3):c.3004C>T | 0.00203 | deleterious | prob damaging | 0.38702 | VUS | Benign | No |
| 641 | MYBPC3 | c.2860G>A, p.Ala954Thr | 11:47335087 |  | 0.00002093 | tolerated | benign | 0.00086 | VUS | VUS | Yes |
| 651 | MYBPC3 | c.1828G>C, p.Asp610His | 11:47341207 | NM_000256.3(MYBPC3):c.1828G>C | 0.00004887 | deleterious | prob damaging | 0.16857 | VUS | VUS | Yes |
| 665 | MYBPC3 | c.1418T>C, p.Phe473Ser | 11:47342869 | NM_000256.3(MYBPC3):c.1418T>C | N/A |  |  | 0.1378 | VUS | Likely pathogenic | No |
| 672 | MYBPC3 | c.2992c>g, p.Q998E | 11:47333924 | NM_000256.3(MYBPC3):c.2992C>G | 0.002428 | deleterious | prob damaging | N/A | N/A | Benign | N/A |
| 706 | MYBPC3 | c.713G>A, p.Arg238His | 1:77929505 | NM_000256.3(MYBPC3):c.713G>A | N/A |  |  | 0.95981 | Path/LP | VUS | No |
| 743 | MYBPC3 | c.565G>A, p.Val189Ile | 11:47349863 | NM_000256.3(MYBPC3):c.565G>A | 0.001911 | tolerated | benign | N/A | N/A | Benign | N/A |
| 745 | MYBPC3 | c.2210C>T, p.Thr737Met | 11:47338618 | NM_000256.3(MYBPC3):c.2210C>T | 0.0001605 | tolerated | prob damaging | 0.00302 | Benign/LB | VUS | No |
| 747 | MYBPC3 | c.529C>T, p.Arg177Cys | 11:47349899 | NM_000256.3(MYBPC3):c.529C>T | 0.00009793 | deleterious | poss damaging | 0.04874 | Benign/LB | VUS | No |
| 789 | MYBPC3 | c.1786G>A, p.Gly596Arg | 11:47341995 | NM_000256.3(MYBPC3):c.1786G>A | 0.00006978 | deleterious | prob damaging | 0.24681 | VUS | VUS | Yes |
| 795 | MYBPC3 | c.2614G>A, p.Glu872Lys | 11:47336000 | NM_000256.3(MYBPC3):c.2614G>A | 0.0001188 | deleterious | prob damaging | 0.08145 | Benign/LB | Likely benign | Yes |
| 887 | MYBPC3 | c.758A>G, p.Asn253Ser | 11:47348438 | NM_000256.3(MYBPC3):c.758A>G | 0.00002794 | tolerated | benign | 0.00695 | Benign/LB | VUS | No |
| 313 | MYH7 | c. 1988G>A p.Arg663His | 14:23426833 | NM_000257.4(MYH7):c.1988G>A | 0.00004898 | tolerated | poss damaging | 0.99059 | Path/LP | Pathogenic | Yes |
| 417 | MYH7 | c.4348G>A, p.D1450N | 14:23417508 | NM_000257.3(MYH7):c.4348G>A | 0.00003488 | deleterious | prob damaging | 0.82799 | VUS | VUS | Yes |
| 94 | MYH7 | c.1804A>G, p.Asn602Asp | 14:23427669 | NM_000257.3(MYH7):c.1804A>G | N/A |  |  | 0.99762 | Path/LP | VUS | No |
| 193 | MYH7 | c.3286G>T, p.Asp1096Tyr | 14:23421008 | NM_000257.4(MYH7):c.3286G>T | 0.0001256 | deleterious | prob damaging | 0.06124 | Benign/LB | VUS | No |
| 207 | MYH7 | c.2155C>T, p.Arg719Trp | 14:23425971 | NM_000257.4(MYH7):c.2155C>T | 0.000006978 | deleterious | poss damaging | 0.99102 | Path/LP | Pathogenic | Yes |
| 209 | MYH7 | c.1012G>A, p.Val338Met | 14:23429901 | NM_000257.3(MYH7):c.1012G>A | N/A |  |  | 0.99175 | Path/LP | Pathogenic | Yes |
| 284 | MYH7 | c.1711G>A, p.Gly571Arg | 14:23427762 | NM_000257.3(MYH7):c.1711G>A | N/A |  |  | 0.80033 | VUS | VUS | Yes |
| 329 | MYH7 | c.746G>A, p.Arg249Gln | 14:23431468 | NM_000257.3(MYH7):c.746G>A | N/A |  |  | 0.99932 | Path/LP | Pathogenic | Yes |
| 338 | MYH7 | c.2167C>T, p.Arg723Cys | 14:23425814 | NM_000257.4(MYH7):c.2167C>T | 0.00002094 | tolerated | prob damaging | 0.99303 | Path/LP | Pathogenic | Yes |
| 343 | MYH7 | c.3464G>A, p.Glyn155Glu | 14:23420107 | NM_000257.3(MYH7):c.3464G>A | 0.000007005 | deleterious | poss damaging | 0.97922 | Path/LP | VUS | No |
| 362 | MYH7 | c.2788G>C, p.Glu930Gln | 14:23424041 | NM_000257.3(MYH7):c.2788G>C | N/A |  |  | 0.99769 | Path/LP | Pathogenic | Yes |
| 396 | MYH7 | c.2302G>A, p.Gly768Arg | 14:23425403 | NM_000257.3(MYH7):c.2302G>A | N/A |  |  | 0.99904 | Path/LP | Pathogenic | Yes |
| 452 | MYH7 | c.2572C>T, p. Arg858Cys | 14:23424876 | NM_000257.3(MYH7):c.2572C>T | 0.00002791 | deleterious | poss damaging | 0.48506 | VUS | Pathogenic | No |
| 479 | MYH7 | c.208A>T, p.Thr70Ser | 14:23433221 | NM_000257.3(MYH7):c.208A>T | N/A |  |  | 0.06566 | Benign/LB | VUS | No |
| 483 | MYH7 | c.920C>T, p.Pro307Leu | 14:23430639 | NM_000257.3(MYH7):c.920C>T | N/A |  |  | 0.90134 | Path/LP | VUS | No |
| 483 | MYH7 | c.1426C>G, p.Leu476Val | 14:23428652 | NM_000257.3(MYH7):c.1426C>G | N/A |  |  | 0.99894 | Path/LP | VUS | No |
| 488 | MYH7 | c.2221G>T, p.Gly741Trp | 14:23425760 | NM_000257.4(MYH7):c.2221G>T | 0.000006978 | deleterious | prob damaging | 0.99838 | Path/LP | Pathogenic | Yes |
| 498 | MYH7 | c.5762G>A, p.R1921Q | 14:23413787 | NM_000257.3(MYH7):c.5762G>A | 0.000006977 | deleterious | poss damaging | 0.95114 | Path/LP | VUS | No |
| 509 | MYH7 | c.2968G>A, p.Ala990Thr | 14:23423678 | NM_000257.3(MYH7):c.2968G>A | N/A |  |  | 0.00474 | Benign/LB | VUS | No |
| 525 | MYH7 | c.1570A>G, p.Ile524Val | 14:23428508 | NM_000257.3(MYH7):c.1570A>G | N/A |  |  | 0.99566 | Path/LP | VUS | No |
| 527 | MYH7 | c.3196A>G, p.I1066V | 14:23422229 | NM_000257.3(MYH7):c.3196A>G | N/A |  |  | 0.00426 | Benign/LB | VUS | No |
| 572 | MYH7 | c.5740G>A, p.Glu1914Lys | 14:23413809 | NM_000257.4(MYH7):c.5740G>A | N/A |  |  | 0.94058 | Path/LP | Likely pathogenic | Yes |
| 596 | MYH7 | c.2609G>A, p.R870H | 14:23424839 | NM_000257.4(MYH7):c.2609G>A | 0.00001396 | deleterious | poss damaging | 0.998 | Path/LP | Pathogenic | Yes |
| 607 | MYH7 | c.1954A>G, p.Arg652Gly | 14:23427242 | NM_000257.3(MYH7):c.1954A>G | 0.000006978 | deleterious | poss damaging | 0.99897 | Path/LP | Pathogenic | Yes |
| 607 | MYH7 | c.2692C>G, p.Leu898Val | 14:23424137 | NM_000257.3(MYH7):c.2692C>G | N/A |  |  | 0.99201 | Path/LP | Likely pathogenic | Yes |
| 614 | MYH7 | c.2711G>A, p.Arg984His | 14:23424118 | NM_000257.3(MYH7):c.2711G>A | N/A |  |  | 0.9906 | Path/LP | Pathogenic | Yes |
| 639 | MYH7 | c.1357C>T, p.Arg453Cys | 14:23429005 | NM_000257.4(MYH7):c.1357C>T | 0.000006977 | deleterious | prob damaging | 0.99811 | Path/LP | Pathogenic | Yes |
| 639 | MYH7 | c.50G>A, p.Arg17His | 14:23433683 | NM_000257.3(MYH7):c.50G>A | 0.00001395 | deleterious | poss damaging | 0.81036 | VUS | VUS | Yes |
| 649 | MYH7 | c.1208G>A, p.Arg403Gln | 14:23429278 | NM_000257.4(MYH7):c.1208G>A | N/A |  |  | 0.99954 | Path/LP | Pathogenic | Yes |
| 651 | MYH7 | c.2722C>G, p.Leu908Val | 14:23424107 | NM_000257.4(MYH7):c.2722C>G | 0.00001395 | deleterious | prob damaging | 0.99932 | Path/LP | Pathogenic | Yes |
| 745 | MYH7 | c.1031A>T, p.Glu344Val |  |  |  |  |  | 0.88448 | VUS | VUS | Yes |
| 765 | MYH7 | c.2359C>T, p.R787C | 14:23425346 | NM_000257.3(MYH7):c.2359C>T | 0.00008378 | tolerated | poss damaging | 0.03849 | Benign/LB | VUS | No |
| 770 | MYH7 | c.77C>T, p.Ala26Val | 14:23433656 | NM_000257.4(MYH7):c.77C>T | 0.0002162 | tolerated | benign | 0.19913 | VUS | Benign | No |
| 774 | MYH7 | c.1331 A>G; p.Asn444Ser | 14:23429031 | NM_000257.4(MYH7):c.1331A>G | N/A |  |  | 0.98866 | Path/LP | Likely pathogenic | Yes |
| 809 | MYH7 | c.1003G>C, p.Ala335Pro | 14:23429910 | NM_000257.3(MYH7):c.1003G>C | N/A |  |  | 0.99729 | Path/LP | VUS | No |
| 809 | MYH7 | c.5500G>T, p.Ala1834Ser | 14:23415054 | NM_000257.3(MYH7):c.5500G>T | N/A |  |  | 0.00519 | Benign/LB | VUS | No |
| 886 | MYH7 | c.4423C>T, p.Arg1475Cys | 14:23417249 | NM_000257.3(MYH7):c.4423C>T | 0.00009075 | deleterious | prob damaging | 0.922488 | Path/LP | VUS | No |
| 394 | MYL3 | c.530A>G, p.E177G | 3:46858413 | NM_000258.2(MYL3):c.530A>G | 0.00002793 | deleterious | poss damaging | 0.9848 | Path/LP | VUS | No |
| 402 | MYL2 | c.401A>C, p.Glu134Ala | 12:110913097 | NM_000432.3(MYL2):c.401A>C | 0.0002865 | deleterious | poss damaging | 0.98883 | Path/LP | VUS | No |
| 484 | MYL2 | c.173G>A, p.R58Q | 12:110914287 | NM_000432.3(MYL2):c.173G>A | N/A |  |  | 0.97737 | Path/LP | Pathogenic | Yes |
| 767 | MYL2 | c.82G>A, p.Glu28Lys | 12:110919115 | NM_000432.3(MYL2):c.82G>A | N/A |  |  | 0.99611 | Path/LP | VUS | No |
| 213 | PRKAG2 | c.620C>G, p.Ser207Cys | 7:151675484 | NM_016203.3(PRKAG2):c.620C>G | N/A |  |  | 0.00098 | Benign/LB | VUS (carrier) | No |
| 319 | PRKAG2 | c.1267C>A, p.Gln423Lys | 7:151565852 | NM_016203.3(PRKAG2):c.1267C>A | 0.0001186 | tolerated | benign | 0.0093 | Benign/LB | VUS | No |
| 508 | PRKAG2 | c.251G>A, p.Arg84Gln | 7:151781367 | NM_016203.3(PRKAG2):c.251G>A | 0.0003562 | tolerated, low confidence | benign | 0.0039 | Benign/LB | Likely benign | Yes |
| 524 | PRKAG2 | c.521C>T, p.Thr174Met | 7:151675583 | NM_016203.3(PRKAG2):c.521C>T | 0.0002023 | tolerated, low confidence | benign | 0.00088 | Benign/LB | VUS | No |
| 733 | PRKAG2 | c.325T>G, p.Ser109Ala | 7:151781293 | NM_016203.3(PRKAG2):c.325T>G | 0.0002443 | tolerated, low confidence | benign | 0.00288 | Benign/LB | VUS | No |
| 816 | PRKAG2 | c.1459T>C, p.Tyr487His | 7:151564203 | NM_016203.3(PRKAG2):c.1459T>C | N/A |  |  | 0.88101 | VUS | Pathogenic | No |
| 333 | PTPN11 | c.1658C>T, p.Thr553Met | 12:112502202 | NM_002834.4(PTPN11):c.1658C>T | 0.0004679 | tolerated | benign | 0.00505 | Benign/LB | Benign | Yes |
| 736 | PTPN11 | c.1492C>T, p.Arg498Trp | 12:112489068 | NM_002834.4(PTPN11):c.1492C>T | N/A |  |  | 0.99028 | Path/LP | Pathogenic | Yes |
| 854 | PTPN11 | c.1391G>C, p.Gly464Ala | 12:112488454 | NM_002834.4(PTPN11):c.1391G>C | N/A |  |  | 0.99287 | Path/LP | Pathogenic | Yes |
| 80 | SCN5A | c.1237G>T, p.Ala413Ser | 3:38606052 | NM_198056.2(SCN5A):c.1237G>T | 0.00002095 | tolerated | prob damaging | 0.32017 | VUS | VUS | Yes |
| 128 | SCN5A | c.103G>A, p.Gly35Ser | 3:38633205 | NM_198056.2(SCN5A):c.103G>A | 0.00003492 | tolerated | benign | 0.0015 | Benign/LB | VUS | No |
| 158 | SCN5A | c.4978A>G, p.Ile1660Val | 3:38551394 | NM_198056.2(SCN5A):c.4978A>G | 0.00006984 | deleterious | benign | 0.96046 | Path/LP | Likely pathogenic | Yes |
| 214 | SCN5A | c.5477G>A, p.Arg1826His | 3:38550895 | NM_000335.4(SCN5A):c.5474G>A | 0.0001676 | tolerated | poss damaging | 0.03042 | Benign/LB | VUS | No |
| 217 | SCN5A | c.3308C>A, p.Ser1103Tyr | 3:38579413 | NM_198056.2(SCN5A):c.3308C>A | 0.000006976 | deleterious | prob damaging | N/A | N/A | Likely benign | N/A |
| 372 | SCN5A | c.2074C>A, p.Gln692Lys | 3:38597917 | NM_000335.4(SCN5A):c.2074C>A | 0.0001814 | tolerated | benign | 0.00144 | Benign/LB | Likely benign | N/A |
| 373 | SCN5A | c.673C>T, p.Arg225Trp | 3:38613773 | NM_000335.4(SCN5A):c.673C>T | 0.00008374 | deleterious | prob damaging | 0.61104 | VUS | Pathogenic | No |
| 374 | SCN5A | c.3905T>C, p.Leu1302Pro |  |  |  |  |  | 0.9582 | Path/LP | VUS | No |
| 476 | SCN5A | c.3250G>C, p.Gly1084Arg | 3:38579474 | NM_198056.2(SCN5A):c.3250G>C | 0.00001396 | tolerated | benign | 0.00229 | Benign/LB | VUS | No |
| 515 | SCN5A | c.262A>G, p.Ser88Gly | 3:38633046 | NM_198056.2(SCN5A):c.262A>G | N/A |  |  | 0.00611 | Benign/LB | VUS | No |
| 580 | SCN5A | c.2989G>A, p.Ala997Thr | 3:38581170 | NM_198056.2(SCN5A):c.2989G>A | 0.0001467 | tolerated | benign | 0.00235 | Benign/LB | VUS | No |
| 608 | SCN5A | c.569G>A, p.R190Q | 3:38620885 | NM_198056.2(SCN5A):c.569G>A | 0.00003489 | deleterious | poss damaging | 0.85893 | VUS | VUS | Yes |
| 652 | SCN5A | c.3269C>T, p.Pro1090Leu | 3:38579455 | NM_198056.2(SCN5A):c.3269C>T | 0.0007607 | tolerated | benign | N/A | N/A | Benign | N/A |
| 784 | SCN5A | c.5038G>A, p.Ala1680Thr | 3:38551280 | NM_198056.2(SCN5A):c.5038G>A | 0.00001397 | deleterious | poss damaging | 0.10418 | VUS | VUS | Yes |
| 819 | SCN5A | c. 5860 G>A, p.Glu1954Lys | 3:38550512 | NM_198056.2(SCN5A):c.5860G>A | 0.0001256 | tolerated | benign | 0.00214 | Benign/LB | VUS | No |
| 186 | TNNI3 | c.575G>A, p.Arg192His | 19:55151892 | NM_000363.4(TNNI3):c.575G>A | N/A |  |  | 0.99913 | Path/LP | Pathogenic | Yes |
| 361 | TNNI3 | c.557G>A, p.Arg186Gln | 19:55151910 | NM_000363.4(TNNI3):c.557G>A | 0.000006978 | tolerated | benign | 0.92336 | Path/LP | Pathogenic | Yes |
| 421 | TNNI3 | c.592C>G, p.Leu198Val | 19:55151875 | NM_000363.4(TNNI3):c.592C>G | 0.00000698 | tolerated | benign | 0.60492 | VUS | Likely pathogenic | No |
| 469 | TNNI3 | c.484C>T, p.Arg162Trp | 19:55154095 | NM_000363.4(TNNI3):c.484C>T | 0.000006995 | deleterious | prob damaging | 0.64308 | VUS | Likely pathogenic | No |
| 507 | TNNI3 | c.244C>T, p.Pro82Ser | 19:55156239 | NM_000363.4(TNNI3):c.244C>T | 0.006847 | deleterious | prob damaging | N/A | N/A | Benign | N/A |
| 522 | TNNI3 | c.458C>A, p.Ala153Asp | 19:55154121 | NM_000363.4(TNNI3):c.458C>A | N/A |  |  | 0.97879 | Path/LP | VUS | No |
| 571 | TNNI3 | c.610C>T, p.Arg204Cys | 19:55151857 | NM_000363.4(TNNI3):c.610C>T | 0.00000698 | deleterious | prob damaging | 0.98887 | Path/LP | Pathogenic | Yes |
| 694 | TNNI3 | c.122T>A, p.Ile41Asn |  |  |  |  |  | 0.52789 | VUS | VUS | Yes |
| 860 | TNNI3 | c.451G>A, p.Ala151Thr | 19:55154128 | NM_000363.4(TNNI3):c.451G>A | N/A |  |  | 0.99647 | Path/LP | Likely pathogenic | Yes |
| 877 | TNNI3 | c.5C>T, p.Ala2Val | 19:55157585 | NM_000363.4(TNNI3):c.5C>T | 0.00000698 | deleterious, low confidence | poss damaging | 0.06644 | Benign/LB | VUS | No |
| 72 | TPM1 | c.91G>A, p.A31T | 15:63042920 | NM_001018005.1(TPM1):c.91G>A | 0.000006977 | tolerated | benign | 0.61398 | VUS | VUS | Yes |
| 221 | TPM1 | c.416A>T, p.Glu139Val | 15:63059604 | NM_000366.5(TPM1):c.416A>T | N/A |  |  | 0.97339 | Path/LP | Likely pathogenic | Yes |
| 287 | TPM1 | c.496G>A, p.Ala166Thr | 15:63060872 | NM_001018005.2(TPM1):c.496G>A | N/A |  |  | 0.99369 | Path/LP | Likely pathogenic | Yes |
| 352 | TPM1 | c.23T>G, p.M8R | 15:63042852 | NM_001018005.1(TPM1):c.23T>G | N/A |  |  | 0.99257 | Path/LP | Likely pathogenic | Yes |
| 368 | TPM1 | c.613A>C, p.Lys205Gln | 15:63061247 | NM_000366.5(TPM1):c.613A>C | N/A |  |  | 0.69923 | VUS | VUS | Yes |
| 476 | TPM1 | c.797A>G, p.Lys266Arg | 15:63064088 | NM_001018005.1(TPM1):c.797A>G | 0.0002024 | tolerated | benign | 0.02364 | Benign/LB | VUS | No |
| 561 | TPM1 | c.602C>T, p.Thr201Met | 15:63061751 | NM_001018005.1(TPM1):c.602C>T | N/A |  |  | 0.27253 | VUS | VUS | Yes |
| 585 | TPM1 | c.574G>A, p.Glu192Lys | 15:63061723 | NM_001018005.1(TPM1):c.574G>A | N/A |  |  | 0.95503 | Path/LP | Pathogenic | Yes |
| 760 | TPM1 | c.829G>A, p.Ala277Thr | 15:63064120 | NM_001018005.1(TPM1):c.829G>A | 0.00009074 | tolerated | benign | 0.01436 | Benign/LB | VUS | No |
| 845 | TPM1 | c.418A>C, p.K140Q | 15:63059606 | NM_001018005.1(TPM1):c.418A>C | N/A |  |  | 0.74302 | VUS | VUS | Yes |
| 29 | TNNT2 | c.268A>G, p.Met90Val | 1:201365289 | NM_001276345.2(TNNT2):c.313A>G | 0.00002094 | tolerated | benign | 0.92236 | Path/LP | VUS | No |
| 49 | TNNT2 | c.415C>T, p.Arg139Cys | 1:201364342 | NM_001001430.2(TNNT2):c.415C>T | N/A |  |  | 0.86219 | VUS | Likely pathogenic | No |
| 250 | TNNT2 | c.421C>T, p.R141W | 1:201364366 | NM_001001430.2(TNNT2):c.421C>T | N/A |  |  | 0.9569 | Path/LP | Pathogenic | Yes |
| 254 | TNNT2 | c.281G>A, p.R94H | 1:201365291 | NM_001001430.2(TNNT2):c.281G>A | N/A |  |  | 0.98161 | Path/LP | Pathogenic | Yes |
| 385 | TNNT2 | c.330T>G, p.Phe110Leu | 1:201365242 | NM_001001430.2(TNNT2):c.330T>G | N/A |  |  | 0.96512 | Path/LP | Pathogenic | Yes |
| 613 | TNNT2 | c.536C>T, p.Ser179Phe | . | NM_001001430.2(TNNT2):c.536C>T | N/A |  |  | 0.8247 | VUS | Pathogenic | No |
| 718 | TNNT2 | c.400c>g, p.R134G | 1:201364357 | NM_000364.3(TNNT2):c.430C>G | N/A |  |  | 0.99512 | Path/LP | Likely pathogenic | Yes |
